# Walking a fine line: is it possible to remain an empathic physician and have a hardened heart?

**DOI:** 10.3389/fnhum.2013.00233

**Published:** 2013-06-11

**Authors:** Bruce W. Newton

**Affiliations:** Department of Neurobiology and Developmental Sciences, University of Arkansas for Medical SciencesLittle Rock, AR, USA

**Keywords:** empathy, vicarious, cognitive, affective, burnout, medical, students, BEES

## Abstract

Establishing an empathic physician–patient relationship is an essential physician skill. This chapter discusses the sexually dimorphic aspects of the neural components involved in affective and cognitive empathy, and examines why men and women medical students or physicians express different levels of empathy. Studies reveal levels of medical student affective or cognitive empathy can help reveal which medical specialty a student will enter. The data show students or physicians with higher empathy enter into specialties characterized by large amounts of patient contact and continuity of care; and individuals with lower levels of empathy desire specialties having little or no patient contact and little to no continuity of care. Burnout and stress can decrease the empathy physicians had when they first entered medical school to unacceptable levels. Conversely, having a too empathetic physician can let patient conditions and reactions interfere with the ability to provide effective care. By learning to blunt affective empathic responses, physicians establish a certain degree of empathic detachment with the patient in order to provide objective care. However, a physician must not become so detached and hardened that their conduct appears callous, because it is still important for physicians, especially those in specialties with a large amount of patient contact, to use empathic communication skills.

## Why is physician empathy important?

How a physician interacts with patients impacts how the patient views the physician. Patients desire an empathic physician who listens and expresses an understanding of their medical condition. Empathy is a highly desirable professional trait, since empathic communication skills promote patient satisfaction, establishes trust, reduces anxiety, increases adherence to treatment regimens, improves health outcomes, as well as decreasing the likelihood of malpractice suits (Butow et al., 1997; Levinson et al., 1997; Roter et al., 1997; Brownell and Coté, 2001; Glaser et al., 2007; Del Canale et al., 2012). A physician may possess competent diagnostic skills, yet be considered by patients as “ineffective” because the physician misses the link between patient satisfaction and adherence to medical instructions and empathy. Being empathic not only benefits the patient, it also has a positive impact upon the physician who can be more effective and provide better care (Di Blasi et al., 2001). Empathic physicians are happier in their workplace, have more enjoyment seeing patients, are less likely to succumb to severe burn-out, and may be more clinically-competent (Suchman et al., 1993; Davis, 1996; Hojat et al., 2002a; Kataoka et al., 2012). Yet, as discussed in section How Physician Stress and Burnout Impacts Empathy, work-related stressors influence how physicians relate to patients.

## What is empathy?

Empathy is a multidimensional trait with many factors contributing to its development and expression (e.g., see Eisenberg, 2005). Empathy is not sympathy or pity where you favor or feel sorry for another, respectively. There have been numerous attempts to define empathy, but embedded in all of the definitions are the concepts that empathy combines aspects of thinking and feeling. Although the distinction can be considered somewhat blurred, empathy can be divided into two main definitions or types: affective (vicarious) and cognitive (imaginative; Engelen and Röttger-Rössler, 2012). Affective empathy is “an individual's vicarious emotional response to perceived emotional experiences of others”; whereas cognitive empathy is “an individual's ability to imaginatively take the role of another so as to understand and accurately predict that person's thoughts, feelings and actions” (Mehrabian et al., 1988). The first definition reflects an innate emotional response, i.e., a “gut reaction,” while the second definition reflects a learned ability to imagine and intellectualize or “role-play.”

In this chapter the term “affective empathy” is equal to vicarious, innate or emotional empathy, and “cognitive empathy” is equal to imaginative empathy or affective theory of mind (ToM).

Regardless of the definition you prefer, a physician has to “feel into” the patient and consider, either emotionally and/or cognitively, the patient is their counterpart in a particular situation. There is no reason to debate if the affective or cognitive aspect of empathy is most important within physicians, since it is how the physician interacts via verbal communication and body language that is important to the patient. Larson and Yao (2005) consider empathy expressed by physicians to be an “emotional labor,” where physicians can either use “deep acting” (i.e., method acting) to generate consistent affective and cognitive reactions to a patient, or “surface acting” to forge empathic behavior in the absence of cognitive or affective reactions to the patient.

Being considered empathetic by the patient makes the physician more sociable and able to engage in meaningful therapeutic interactions benefiting both the patient and the physician. This becomes especially important when physicians have to correctly interpret facial or non-verbal expressions of pain behavior (Goubert et al., 2005). To do this, the physician needs to reflect, via perspective-taking, upon their vicarious empathic state, orchestrated by more primitive brain regions (e.g., insula, anterior cingulate cortex and amygdala), and then make an appropriate emotional response (Hadjistavropoulos et al., 2011). As discussed later, neocortical regions modulate the vicarious feelings, e.g., the prefrontal cortex (PFC) and temporoparietal junction (Lamm et al., 2007).

## Empathy scales reveal sexual dimorphism

Using the Balanced Emotional Empathy Scale (BEES; Mehrabian, 1996), the degree of affective empathy has been shown to consistently differ between the sexes with women having higher BEES scores, i.e., showing greater degrees of affective empathy, than men (Mehrabian et al., 1988; Newton et al., 2000, 2008a,b; Shapiro et al., 2004; Dehning et al., 2012). The Jefferson Scale of Physician Empathy (JSPE; Hojat et al., 2001), which measures cognitive empathy, gives variable results on whether there is a consistent female > male sex difference (Hojat et al., 2002a,b; Kataoka et al., 2009; Rahimi-Madiseh et al., 2010; Beattie et al., 2012; Suh et al., 2012). Other scales that measure cognitive empathy show women generally report higher levels of empathy than men (Diseker and Michielutte, 1981; Mestre et al., 2009; Neumann et al., 2011; Dehning et al., 2012). This chapter will focus on studies using the BEES and the JSPE. Regarding any survey instrument, there is the caveat that the BEES and JSPE only reveal the self-reported “trait empathy,” which can differ from the “state empathy” representing the actual affective or cognitive state of mind expressed during a specific encounter.

## Empathy, prosocial behavior, and moral development

Although there is some debate on how empathy contributes to prosocial behavior, the consensus is prosocial behavior is linked to, or augmented by, empathy (Singer and Lamm, 2009). Studies by Eisenberg and colleagues have confirmed the link between empathy and the willingness to help others (Eisenberg and Fabes, 1990; Eisenberg, 2005, 2007). An individual who exhibits a high degree of prosocial behavior as a young child, will continue to exhibit prosocial behavior as a young adult (Eisenberg et al., 1999; Eisenberg, 2005)—the age at which most people enter into undergraduate medical education.

Moral reasoning is correlated with empathy, because those individuals who display empathy-related responding (even at pre-school age) show a higher level of moral reasoning and reduced use of hedonistic reasoning as adults (Eisenberg et al., 1991; Eisenberg, 2005, 2007). Being a physician demands a high degree of moral judgment, yet medical school can stunt moral growth and increase cynicism (Self et al., 1993; Feudtner et al., 1994; Hafferty and Franks, 1994; Testerman et al., 1996; Patenaude et al., 2003). Accordingly, numerous studies have shown the erosion of physician affective and cognitive empathy, a decrease in numerous attitude measurements, and an increase in derogatory remarks and cynicism toward patients which can be exacerbated after entering clinical rotations, residencies or the workforce (Testerman et al., 1996; Bellini et al., 2002; Griffith and Wilson, 2003; Woloschuk et al., 2004; Dyrbye et al., 2005; Newton et al., 2008a,b; Hojat et al., 2009). This erosion can have a negative impact on both the physician and patient if the physician dislikes the patient and displays unprofessional behavior. An example of professional behavior erosion would be the frustration a physician feels who has repeated interactions with a non-compliant patient who is compromising their health by not adhering to the physician's advice. Thus, if a certain degree of empathy is not inherently present, the physician may not have the ability to suppress their true negative emotions in order to rationally and calmly, once again, explain the need for the non-compliant patient to practice a healthier life-style. (As discussed in the next section, there is a large cognitive component via higher CNS centers used to modulate the initial, vicarious empathic response.) Therefore, low levels of empathy can lead to a decreased ability to respond to others in distress in an appropriate emotional fashion, and to externalize and verbalize problems (Hastings et al., 2000; Zhou et al., 2002). The advantage of being empathetic and prosocial is that it reduces and/or inhibits aggressive actions toward others (Mehrabian and Epstein, 1972; Miller and Eisenberg, 1988).

The ability to express prosocial behavior and empathic concern, ostensibly reducing aggressive interactions, is not restricted to humans. A review of several studies show rodents respond in a prosocial fashion to another's distress (Mogil, 2012); emphasizing this ability is an evolutionarily conserved positive trait. It is interesting to note that much like humans, where women report higher degrees of empathy than men (Mehrabian et al., 1988; Newton et al., 2008a,b), female rats were much more likely to release a trapped cage mate than male rats (Bartal et al., 2011).

## Affective and cognitive empathic responses use different CNS sites

Over the past several decades considerable research has been devoted to elucidating the central nervous system (CNS) sites activated during empathic responses to various controlled situations—especially reactions to pain paradigms. Several recent, excellent review articles (e.g., Singer, 2006; Decety, 2011; Bernhardt and Singer, 2012; Walter, 2012) go into detail about empathy-activated CNS sites. However, a brief review of the different sites involved in affective vs. cognitive empathy, and how this relates to the sexually dimorphic empathic response, is provided.

Studies measuring affective empathy (Fan et al., 2011; Lamm et al., 2011; Bernhardt and Singer, 2012; Walter, 2012) have shown the anterior insula (AI) and the anterior and dorsal mid-cingulate cortex are the most consistently activated sites. Other sites include the inferior frontal gyrus (IFG), amygdala, periaqueductal gray (PAG), and the secondary somatosensory cortex. Affective empathy sites differ from those used for ToM and cognitive empathy, which include the temporoparietal junction, superior temporal sulcus, dorsomedial PFC, ventromedial PFC, and the posteromedial parietal cortex.

Walter (2012, see Figure 1) proposes the existence of a “low road and a high road” to empathy. The low road corresponds to affective empathy where there is an automatic (i.e., visceral) response to the state of another, especially when pain or suffering is being observed. The low road for affective empathy uses the AI, mid-cingulate cortex, amygdala, secondary somatosensory cortex, and the IFG, with the AI and mid-cingulate cortex most consistently activated. These affective empathy sites utilize different portions of the CNS than the high road that corresponds to cognitive ToM. Cognitive ToM uses the temporoparietal junction, superior temporal sulcus, dorsomedial PFC, and posteromedial cortex. Both affective and cognitive ToM pathways communicate with each other via the ventromedial PFC which enables the cognitive empathic expression. Therefore, the ventromedial PFC appears to be the linchpin where crosstalk and processing of CNS inputs from the cognitive ToM and affective empathy regions are combined for the modulation of the cognitive empathic response to the emotional state of the other. Evidence that the ventromedial PFC is responsible for the expression of cognitive empathy comes from patients with ventromedial PFC lesions who have an impairment of expressing cognitive empathy, yet are still able to complete cognitive ToM tasks (Shamay-Tsoory and Aharon-Peretz, 2007). Another study showed patients with a ventromedial PFC lesion had impaired cognitive but not affective empathy measures, whereas the opposite was found for patients with an IFG lesion who had lower affective but not cognitive empathy scores (Shamay-Tsoory et al., 2009).

The high and low roads for the expression of empathy are similar to the “bottom-up vs. top-down” neural processing that occurs for empathic expression (see Decety and Lamm, 2006; Singer and Lamm, 2009). The bottom up, affective empathy can be modified by top-down cognitive ToM informational processing for the generation of the cognitive empathic response. To have a cognitive empathic response the observer must use higher CNS processing, via cognitive ToM regions, to put what the other is going through into an emotional context. This cognitive empathic reaction to the situation of another can then influence an affective empathic response, and vice versa, an initial affective empathic response actives higher CNS regions modulating cognitive empathy. Therefore, the affective aspect of empathy can be modified by higher order executive functioning to make the individual less dependent on their affective empathy inputs.

### Genetic conditions and lesion studies substantiate different CNS regions are used to express affective and cognitive empathy

The above section revealed that different CNS regions are used to express either affective or cognitive empathy. As further proof, a number of studies [along with the aforementioned lesion studies by Shamay-Tsoory et al. (2009) and Shamay-Tsoory and Aharon-Peretz (2007)] have examined how the expression of empathy is altered in individuals who have suffered various CNS lesions. An fMRI study by Danziger et al. (2009) showed individuals with the rare condition of congenital insensitivity to pain still have affective and cognitive CNS regions responding to observed pain, even though these individuals have never felt pain themselves. Observed pain activated the anterior mid-cingulate cortex and the AI in both congenital insensitivity to pain patients and control individuals. The study also showed that BEES scores (measuring affective empathy) in the congenital insensitivity to pain group was significantly, positively correlated with the activity of the ventromedial PFC and anterior cingulate cortex. Danziger et al. (2006) also showed the posteroventral cingulate cortex of the congenital insensitivity to pain patients was significantly correlated with BEES scores when examining facial expressions of pain, such that the stronger the CNS activity for observing pain, the higher the BEES score. Therefore, the intensity of their empathic response was correlated with their degree of affective empathy. In contrast, the control group showed no correlation between the facial expressions and BEES scores. These studies reveal affective empathic behavior can be expressed even when a person has not directly experienced the pain of another. Therefore, physicians should have the ability to “feel into” and have an affective empathic response for patients in pain, and for patients on whom they will inflict pain or prescribe a painful procedure, even though they have not experienced that pain themselves.

Patients who have had traumatic brain injuries (TBI), which involve prefrontal regions and their connections to the limbic system, have changes in cognitive and affective empathy. In a study by Wood and Williams (2008), TBI patients showed twice as many low affective empathy scores when compared to controls. The data revealed men had lower BEES scores than women, and women with TBI had significantly more low BEES scores than the normal female population. Interestingly, there was no relationship between the severity of the TBI and BEES scores. Thus, even a minor head injury can alter affective empathy as much as a more severe TBI.

### Sexual dimorphism for pain plays a role in affective empathy sexual dimorphism

Because observing pain in others elicits an empathic response, Lamm et al. (2011) performed a meta-analysis to determine the empathic cortical regions used when observing pain in others. Results show the bilateral AI and the anteromedial and posteroanterior cingulate cortical regions are consistently activated when observing pain; importantly these same regions are also activated when the observer is experiencing pain themselves. A review by Bernhardt and Singer (2012) indicates the AI and the anterior and mid-cingulate cortex are involved in eliciting the affective empathic response to pain, and these same regions also receive afferents carrying nociceptive information. Therefore, the expression of affective empathy is linked to the pain axis/matrix.

The pain axis/matrix involves CNS regions bringing nociceptive inputs from the periphery to higher cortical regions to be perceived as pain. This axis includes afferents sending nociceptive information into the dorsal horn of the spinal cord or the trigeminal nucleus. The nociceptive information is sent to the thalamus to be relayed to the postcentral gyrus. The thalamus also sends nociceptive afferents to the insular and anterior cingulate cortex, the IFG and PAG: areas processing the affective components of pain and the same regions already implicated in affective empathy (Rainville, 2002; Singer et al., 2004).

In rats, portions of the pain axis are sexually dimorphic. In the spinal cord, the dimorphism extends from the numbers of dorsal root ganglion neurons sending afferent information into the dorsal horn (male > female) to the qualitative and quantitative amounts of various neurotransmitters and receptors used to relay nociceptive inputs to the spinal cord or thalamus (Newton et al., 1990; Newton, 1992; Mills and Sengelaub, 1993; Newton and Tate, 1996; Phelan and Newton, 2000). In this regard, male rats have more of the neurotransmitters to suppress nociception within the spinal cord than female rats (e.g., enkephalin and galanin); whereas, there is no sexual dimorphism for the neuropeptides involved in sending nociceptive inputs into the spinal cord (e.g., substance P and calcitonin gene-related peptide).

The sexual dimorphism has now been shown to extend to regions involved in affective empathy. For example, the PAG has extensive connections with the insular cortex, medial PFC, anterior cingulate cortex, and amygdala (Linnman et al., 2012). Human fMRI studies show sex differences exist in the activation of various cortical regions involved with affective empathy, such that men have a greater PAG connectivity to the insula and PFC than women, and women have a greater PAG connectivity to the mid-cingulate cortex than men (Kong et al., 2010). Other studies have shown men have greater pain-induced activation of the insular cortex than women; whereas women have a greater activation of the medial PFC (Derbyshire et al., 2002; Straube et al., 2009).

Somatic or visceral nociceptive inputs also activate the autonomic nervous system (ANS), and a recent study has shown sex differences in the parasympathetic response of the amygdala, with women having a greater activation than men (Nugent et al., 2011). The ANS connections with the amygdala, insula, and anterior cingulate cortex are well known and these regions are activated in a sexually dimorphic fashion during highly emotional situations (Critchley, 2005). Therefore, the affective component of empathy recruits the same brain regions involved in the cortical modulation of the ANS. For example, the sympathetic activation of the AI and cingulate cortex is characteristic of the activation of these regions by painful stimuli and strong emotions (Singer et al., 2004; Critchley, 2005). Indeed, the representation of autonomic and visceral responses, especially within the right AI, causes the autonomic inputs to become consciously available in order to influence emotional empathic reactions. Further proof the ANS is involved in empathy is pupil size varies when viewing sad faces. Those individuals with higher empathy scores have a greater pupillary response than individuals with lower empathy scores (Harrison et al., 2007). Also, individuals with primary autonomic failure have significantly attenuated BEES scores as compared to age and gender-match controls (Chauhan et al., 2008).

## How does the physician respond to pain and disparate traits in their patients?

How is a physician, who is supposed to have an empathic connection with the patient, respond to the pain being described by the patient, or to the pain they will inflict with a medical procedure? How does the physician deal with the non-compliant patient, where the physician feels the patient will not follow directions; or a patient who is culturally, morally or ethnically different than them? Some physicians have been known to call difficult patients as “heartsink patients,” a descriptive term that accurately describes the unempathetic response physicians have toward these patients (McDonald and O'Dowd, 1991).

Many times a patient comes to a physician because of pain, or a physician has to perform or prescribe interventions that may be painful. The study by Singer et al. (2004) showed that when a painful stimulus was applied to another person, the affective component of pain was activated in the observer, especially the bilateral AI and rostral cingulate cortex. Furthermore, a person will have an even stronger cortical response to another's pain if they have experienced the pain themselves (Lamm et al., 2010). Therefore, how does a physician cope with the pain of others and not become too empathetic which can lead to compassion fatigue, ineffective care, stress, and anxiety (Figley, 2002; Dyrbye et al., 2005; West et al., 2006; Pejušković et al., 2011)? For example, will a surgeon who performs painful procedures on patients be better able to perform the surgery if they have a reduced amount of affective empathy as compared to a family or internal medicine physician who does not perform as many, or as severe, painful procedures? Research may shed light on this question. CNS regions used to elicit empathic responses differ according to whether the observer is looking at facial expressions, which displays emotional-communicative information, vs. the limbs (Gu and Han, 2007; Han et al., 2009; Vachon-Presseau et al., 2012). Perhaps physicians who are in specialties with high amounts of patient contact, e.g., family practice and internal medicine, who are constantly looking at the patient's facial expressions, may have a greater empathic response than physicians who perform painful procedures, e.g., general surgeons or orthopedists, but do not have to look at the patient's face while performing surgery. Indeed, the ability to detect the intensity of another's pain is most highly correlated with the degree of the facial response of the one in pain (Gu and Han, 2007; Saarela et al., 2007; Han et al., 2009; Hadjistavropoulos et al., 2011).

In 2007, Cheng et al. showed physicians who are experts at practicing acupuncture keep a detached perspective while performing a procedure they know causes pain to the patient. Compared to novice physicians and controls, there was a significantly reduced activation of the AI, anterior cingulate cortex and PAG; but an increased activation in the medial and superior PFC and the temporoparietal junction in the expert physicians. These data suggest expert physicians are using cortical regions involved in emotion regulation and ToM to suppress the affective empathy pathway associated with the pain matrix. Furthermore, the expert physicians used significantly lower ratings on the visual analog pain intensity scale for the pain they were inflicting on their patients than the novice and control participants. These results were verified by Decety et al. (2010) who showed internal medicine physicians, in contrast to control participants, used cortical regions controlling executive functions and self-regulation, i.e., dorsolateral and medial PFC and temporoparietal junction, to inhibit the activation of the empathic pain matrix involving the dorsal anterior cingulate cortex, AI, and PAG. Once again, these physicians rated painful stimuli as significantly less painful than the controls. Both of these studies show a clear blunting of the physician's affective empathy by executive cortical regions.

In 2008, Han et al. and Fan and Han (2008) showed a sex difference in the empathic response to observing pain using event-related brain potentials. Their studies showed both men and women have a short-latency empathic response over the frontal lobe to seeing painful pictures, but a long-latency empathic response over central-parietal regions. Placing these data in the context of physicians shows three things. First, there are two CNS responses to empathy, a short-latency response corresponding to affective empathy, and a long-latency response that underpins the later cognitive empathic response to pain in others. Therefore, feeling into the patient occurs first and elicits an affective empathic response, which is then cognitively modified. Second, although there was no sex difference in the short-latency CNS regions activated by affective empathy, only women showed a strong positive correlation between the activation of these regions with their subjective rating of pain in others. Men showed no such correlation. Thus, the degree of the affective empathic response in women is more strongly determined by the degree to which they subjectively feel how much the patient is suffering. Third, the sex difference in the long-latency, cognitive empathic response suggests women have stronger top-down attentiveness in controlling their affective empathy than men; i.e., women physicians evaluate the painful condition of the patient more intensively than male physicians.

The studies by Han et al. (2008) and Fan and Han (2008) were expanded by Decety et al. (2010) who showed a distinct top-down regulation of the affective empathic response in physicians. This top-down (high road) regulation serves to inhibit the bottom-up, affective perception of pain in others via modulation of the PAG by the anterior cingulate cortex (Valet et al., 2004). Since men have a greater number of connections from the insular cortex and PFC to the PAG than women (Kong et al., 2010); this suggests men may have a greater capacity to blunt affective inputs from the PAG than women.

The above studies indicate experienced physicians are using cognitive processes to modulate the affective component of empathy. However, this begs the question if a novice, i.e., a medical student or beginning resident, has the emotional capacity to engage the neural mechanisms to promote detached concern? If not, they may become emotionally over-involved when feeling into the patient, leading to a potential deterioration of effective patient management. This is especially concerning since the PFC does not reach maturity until the mid-20s (e.g., Sowell et al., 1999), and many medical students begin their medical training in their early 20s when they are expected to empathically reassure worried patients (Epstein et al., 2007). The sex differences in the neural processing of empathy for pain (Han et al., 2008) may confound the ability for both male and female physicians to reach an equivalent level of detached concern, yet still use cognitive (role-playing) empathy to maintain effective physician–patient communication. Thus, will the innate amount of affective empathy possessed by a medical student impact how they will communicate with patients, and even determine if they want to be in a specialty having a large degree, or almost no, patient contact?

### The physician and the non-compliant patient

Regarding a non-compliant patient, an inference can be made to the study by Singer et al. (2006) where they evaluated the perceived fairness of others by using the Prisoner's Dilemma Game. For both men and women there was no sexual dimorphism in the activation of brain regions corresponding to affective empathy (anterior cingulate cortex, AI, and PFC). For both sexes, the more empathic the person, the greater the fMRI activation of the aforementioned regions. However, a sex difference was observed when the Prisoner's Dilemma Game was carried out with an “unfair” person. In this instance, men had significantly reduced empathy-related responses when observing an unfair person receiving pain; however, this reduction was not seen in the women observers. Thus, women showed no significant difference when comparing the results between painful trials for fair or unfair individuals. In the context of medicine, this infers male physicians may not be as empathic toward an “unfair,” non-compliant patient as female physicians. Indeed, the 2006 Singer study showed men had an increased activation of brain reward regions correlated with a desire for revenge. This suggests male physicians, especially those with low empathy, may not treat the non-compliant patient as effectively as female physicians or male physicians with higher empathy. Less effective care may be provided in order for the physician to feel the self-satisfaction the non-compliant patient is responsible for their own misery/decreased health by ignoring medical advice (Squier, 1990).

### How do physicians respond to disparate patients?

How do physicians empathically-relate to individuals who are disparate from themselves, e.g., those of a different race or culture or, e.g., the morbidly obese? Physicians who feel angry with patients and yet find these feelings unacceptable, face barriers on how to relate to the patient's perspective. A study by Lamm et al. (2010) demonstrates an observer looking at a person who is responding in a painful, but incongruent fashion to a harmless stimulus (touching the hand with a Q-tip) activates the same empathic neural regions involved with feeling the pain themselves, i.e., bilateral AI, medial, and anterior cingulate cortex. In contrast, a procedure that would be considered painful for the observer, but not for the patient, recruited CNS regions involved with the self-other distinction and ToM cognitive control, e.g., dorsomedial PFC and right inferior frontal cortex (Mitchell et al., 2006; Decety and Lamm, 2007). These studies indicate physicians should have the cognitive ability to adopt the perspective of a patient dissimilar to themselves and communicate in an empathic fashion. But the ability to do so depends upon the recruitment of CNS regions controlling the affective component of empathy. Therefore, the response to pain in others not like ourselves depends upon the top-down regulation of the bottom-up routes of empathy (e.g., Decety and Lamm, 2006; Lamm et al., 2008). This top-down adaptability enables the physician to understand and emote to the feelings of a patient who is in a situation the physician has not experienced, e.g., a female physician empathizing with a male patient reluctant to have a rectal exam, or any physician relating to someone who has suffered seizures or broken bones, when they themselves have never experienced these traumatic events.

The above positive aspects of a physician being able to establish an empathic relationship with the disparate patient need to be tempered with other results. Can physicians reliably empathize with patients toward whom they naturally feel little or even negative emotions, when it has been shown that empathic responses in the anterior cingulate cortex and AI are influenced with perceived group membership and racial bias? The activation of these empathic regions are reduced when the person observes others different than themselves (Xu et al., 2009; Avenanti et al., 2010; Hein et al., 2010). Therefore, a physician has to be consciously aware of any bias within themselves, e.g., negative feelings for obese patients (Huizinga et al., 2009), and be prepared to cognitively inhibit the affective empathic bias. This becomes especially important when dealing with patients in pain. A study by Drwecki et al. (2011) showed empathy played a role in the quality of pain treatment nurses offered to African Americans or European Americans, such that African Americans received less effective pain management. On a positive note, the study suggested “perspective-taking” intervention could be used to help ameliorate the treatment disparities (see Batson et al., 1997). Therefore, incorporation of this technique into student and physician training can make them aware of this inherent nature to discriminate.

Considering that most physicians participate in a health care team when dealing with patients, it becomes important to question whether interactions with team members who are more empathetic than the physician can influence the physician's behavior. Three examples of increasing prosocial behavior include a study by Drwecki et al. (2011) who showed nurses were more empathetic toward patients in pain, regardless of race, than controls. Another was third year medical students watching exemplary team behavior in the operating room. This made the students more aware of the need to comfort patients and to cooperate and respect other healthcare professionals (Curry et al., 2011). Finally, “human factors” training during surgical clerkships resulted in students being more likely to ask a nurse's perspective on an action plan and increased student–patient communication (Cahan et al., 2010).

## Do sexually dimorphic levels of affective and cognitive empathy determine what medical specialty a student will select?

Although many studies demonstrate sex differences in affective and cognitive empathy among medical students or physicians, few studies have examined medical student empathy changes over time, or have correlated levels of empathy with student or physician specialty choice. Elucidating how empathy is involved with specialty choice becomes important when examining the correlation of empathy with medical student or physician coping skills and the stress of treating patients who are in pain.

Several past studies have suggested certain personality traits of medical students can be used to help predict what medical specialty the student will practice (Rezler, 1974; Hojat et al., 1998; Batenburg et al., 1999). The recent longitudinal affective empathy study by Newton et al. (2008a,b), which surveyed the 2001–2004 graduating classes at the University of Arkansas for Medical Sciences, clearly showed affective empathy levels can indicate what specialty a medical student desires to practice. During the longitudinal empathy study, medical students selected, out of a possible 23 choices, what specialty they would like to enter each time they took the BEES. Newton et al. (2000, 2008a,b) broke the specialties into two different classifications: “core” and “non-core.” There are five core specialties, each characterized by a large degree of patient contact and continuity of care: family and internal medicine, general pediatrics, obstetrics and gynecology (Ob/Gyn), and psychiatry. Non-core specialties (e.g., radiology, emergency medicine, anesthesiology, pathology, surgery) are characterized by low or no patient contact and little or no continuity of care.

At the beginning of the senior year, students with the highest BEES scores desired to enter the core specialties vs. those students with lower BEES scores who desired to enter the non-core specialties. These data can be further broken down by gender. After completing the first three years of undergraduate medical school, women who wanted to enter core specialties had a 13.0% drop in BEES scores compared to their BEES score obtained during orientation to medical school (i.e., base line data). Yet women who desired to enter non-core specialties had more than a two-fold larger drop in BEES scores (29.3%) compared core women. By the start of the senior year, core-selecting men had a 25.8% reduction in BEES scores, and non-core men dropped by 38.7%. All of these declines are significantly different from the BEES scores obtained during freshman orientation. These data show students who desire to enter core specialties with a large amount of patient contact and continuity of care better maintain their affective empathy than students who want to enter non-core specialties, and the rate of decline in core BEES scores was half that of their non-core classmates.

It is interesting to note the largest drops in BEES scores occurred after the completion of the first basic science year of medical school and the first year of clinical rotations (Newton et al., 2008a). It was hypothesized a drop in BEES scores would occur after completing the first basic science year of medical education, and the authors suggested the reason is the students may be suffering from traumatic deidealization (Kay, 1990). The drop in BEES scores after finishing the first year of clinical rotations was unexpected. The authors had expected BEES scores of third year (junior) students to either stay stable or rise because the students were obviously excited about being finished with “book work” and could now start clinical rotations and see patients. The significant drop in affective empathy while seeing patients was disconcerting, since the students were supposed to be learning how to establish an empathic physician–patient relationship rather than decreasing their affective empathy. The drop in affective empathy levels after completing the first year of clinical rotations may be attributed to the severity of cases seen in a tertiary care hospital and/or the lack of positive physician role models. An ongoing analysis of the above data (Newton et al., 2008a) suggests students with high freshman BEES scores, who say they desire to enter non-core specialties, shift to selecting core specialties by the time they take the BEES at the beginning of their senior year. The opposite is true for students who have low BEES scores obtained during orientation and want to enter a core specialty; they tend to shift to non-core specialties (manuscript submitted).

A study using the JSPE to look at specialty preference in relation to cognitive empathy (Hojat et al., 2005) gave results similar to the BEES data (Newton et al., 2000, 2008a,b). This study showed that freshmen medical students who desired to enter primary care specialties (e.g., family and internal medicine, and pediatrics) scored higher on the JSPE than students who wanted to enter technology- or procedure-based specialties (e.g., orthopedics, ophthalmology, radiology, pathology, neurosurgery). Their results showed no sex differences in cognitive empathy scores when compared to desired medical specialty. Results from this study were confirmed by two other studies (Tavakol et al., 2011; Chen et al., 2012).

Hojat et al. (2002a) also examined physician cognitive empathy using the JSPE. Physicians in psychiatry had the highest JSPE scores, but they were not significantly higher than physicians in internal medicine, pediatrics, emergency medicine, and family medicine. Physicians with the lowest JSPE scores were in orthopedic surgery, neurosurgery, radiology, and anesthesiology. The JSPE data showed no sex differences among the physicians. The difference between the BEES and JSPE results may be a reflection of the two different types of empathy being measured, or that the BEES data came from medical students, whereas the JSPE was used to survey physicians.

### Affective empathy vs. residency match

It is telling when BEES scores, obtained at the beginning of the senior year of undergraduate medical school, are compared to the medical specialty the students actually entered upon graduation (Newton et al., 2008b). For specialties with an *n* ≥ 7 graduates, the BEES scores of the five core specialties ranked in the top six specialties. In rank order, they were Ob/Gyn, general pediatrics, psychiatry, family medicine, anesthesiology (a non-core specialty), and internal medicine. Even though senior BEES scores were lower when compared to the BEES scores obtained during freshman orientation to medical school (vide supra), each of the core specialties still maintained an “average” amount of affective empathy when compared to the normal population. (The average rating is equivalent to the 50th percentile on the bell-shaped curve of BEES scores; Mehrabian, 1996). Therefore, senior students who better maintained their BEES scores, and by inference had the smallest decreases in affective empathy, matched into the core medical specialties characterized by a large degree of patient contact and continuity of care. Almost all non-core specialties had BEES scores lower than the population norm. The non-core specialties ranked as having “slightly low” affective empathy (31st percentile; −0.5 s.d.) were, in descending order of BEES scores, diagnostic radiology, medical pediatrics, ophthalmology, general surgery, urology, and emergency medicine. Non-core specialties ranked as “moderately low” (16th percentile, −1.0 s.d.) were students entering into pathology and orthopedic residencies.

There were several specialties where the number of students who entered them was low enough (*n* ≤ 6) that only a trend average could be established. Graduates entering into dermatology, radiation oncology, and physical medicine residencies had an “average” BEES score; while preventive medicine and nuclear medicine were rated as “moderately low.” Otolaryngology ranked as “very low” (7th percentile; −1.5 s.d.), and plastic surgery and neurosurgery were ranked as “extremely low” (2nd percentile; −2.0 s.d.). The only specialty to rank above “average” was neurology, which was “slightly high” (69th percentile, +0.5 s.d.). (A possible reason for the slightly high BEES score for entering neurology residents is that several of our neurologists are outstanding role models, have won “Humanism Awards” and have a large teaching role.)

The above affective empathy data suggest medical students are self-selecting their specialty choice according to their intrinsic level of affective empathy. Thus, students with the higher BEES scores, who enter into core specialties with a large degree of patient contact and continuity of care, may demonstrate a better bedside manner than those students entering into non-core specialties with little patient contact. In other words, students with higher BEES scores may maintain more of their innate ability to more effectively communicate with their patients in an empathic fashion than those students who select specialties with little patient contact. (This is not to say that all physicians, regardless of their specialty, need to practice empathic communication skills.) It appears the students are aware of their own innate level of affective empathy and enter into the specialties where they are most comfortable with the level of patient contact. Anecdotally, we all either know, or have heard, about physicians in certain specialties having a more brusque bedside manner than physicians in other specialties. The affective empathy study by Newton et al. (2008b) provides some empirical data to support the anecdotal observations, since graduates entering into surgical specialties (general surgery, orthopedic surgery, neurosurgery, plastic surgery) have affective empathy scores 0.5–2.0 s.d. lower than the population mean. Other studies support the observation surgical specialties may have a preponderance of less empathic physicians (Hall et al., 2002; Levinson et al., 2006; Duberstein et al., 2007). However, because women generally have better physician/patient skills than men (Bylund and Makoul, 2002; Mast et al., 2007) and higher BEES scores (Newton et al., 2000, 2008a,b), and because more women are entering surgical specialties formerly dominated by men, the decreased level of affective empathy displayed by physicians in these surgical specialties may be improved by the recent increased presence of normally more empathic women.

Related to the above suggestion, various interventions have helped to increase physician prosocial behavior by learning to respect members of a health care team (many of which are women) and to improve communication skills with team members and patients (Cahan et al., 2010; Curry et al., 2011). However, do these interventions have the same degree of success on all the various specialty fields? The aforementioned studies focused on operating room interactions; yet most interactions take place outside the operating room. Is it possible the cognitive modulation of the vicarious physician empathy can be influenced with whom they interact? To what extent does emotional contagion (see Singer, 2006) and mirror neurons in humans (Baird et al., 2011) play a role in a physician's ability to react in a more empathic, prosocial fashion? These questions become even more complicated with the sex differences in the human mirror-neuron system (female > male for pars opercularis and inferior parietal lobe volumes; Cheng et al., 2009). It remains to be determined if a physician can become more empathic if surrounded by team members displaying empathic behavior, and if women will potentially have a greater positive response than men.

## How physician stress and burnout impacts empathy

Recent studies clearly show being a medical student, resident, or physician is stressful (Dyrbye et al., 2006; West et al., 2006; Nettleton et al., 2008; Pejušković et al., 2011), and women generally have a more adverse response to medical profession stressors than men (Lloyd and Gartrell, 1981; Hojat et al., 1999; Lindfors et al., 2009; Backović et al., 2012). Some degree of stress is found in any profession, and a certain amount of stress can be motivating for some individuals, but physicians exhibit greater burnout from stressors than the general population (Shanafelt et al., 2012). The stressors include, among other things, workload, exposure to patient death/suffering, ethical conflicts, the hidden curriculum and poor role models (e.g., Hafferty and Franks, 1994; Figley, 2002; Dyrbye et al., 2005; Haglund et al., 2009). These stressors, if not managed adequately by the medical student or physician can lead to substance abuse, suicide, increased cynicism, medical errors, impaired competency, burnout, depression, a sense of lack of accomplishment, as well as influencing specialty choice (Dyrbye et al., 2005, 2006; West et al., 2006; Pejušković et al., 2011). Additional studies show cognitive and affective empathy are blunted by these stressors (West et al., 2006; Thomas et al., 2007; Koehl-Hackert et al., 2012). Taft et al. (2011) reveal there is a sexual dimorphism in the strategies used to address stress and burnout. Women use more emotion-based coping skills, whereas men use more problem-focused skills. Over reliance on emotional coping skills was a significant predictor of increased psychological distress and decreased self-efficacy.

Stress can exacerbate emotional responses. Over arousal due to an excessive affective empathic response tends to make a person self-focus and experience personal distress (Wood et al., 1990a,b). A physician's excessive empathic response to a patient can decrease their ability to care for the patient, because the physician focuses on their own vicarious response to the patient's medical situation vs. being attentive to the needs of the patient. So, a physician who is predisposed to becoming overly empathetic to negative situations needs the ability to control their empathic response in order to remain effective. There are two ways a person can become empathically over-aroused: either by the temperament they are born with, which modulates the intensity and quality of their empathic response, or their ability (or inability) to self-regulate their empathic/emotional response. The latter has been termed “effortful emotion-related regulation” where a person modulates the intensity and duration of their expressed emotional behavior in order to accomplish their goals (Eisenberg and Morris, 2002). This emotion-related regulation involves effort, where the person deliberately down-regulates their negative emotions and activates appropriate behavior toward another, even if they really don't want to do so. Yet, the capacity to control temperament and emotional responses varies with the individual. Thus, the temperament of the individual, along with their ability to regulate their emotions contributes to individual differences in empathic capabilities (Eisenberg, 2005). Therefore, a physician needs the ability shift attention away from negative affective inputs they are truly feeling and express their empathic response to the patient in an adaptive manner. This inhibiting mechanism involves the anterior cingulate gyrus which is involved in affective empathy (Rothbart and Bates, 1998). Individuals who have more executive control over cognitive functions should be better able to control their empathic response and less likely to experience personal distress and depression when compared with people who have less executive control over their empathic response (Zalewski et al., 2011).

It is revealing when one compares the rate of physician burn out with trait empathy via BEES and JSPE scores (Hojat et al., 2002b; Newton et al., 2008b; Shanafelt et al., 2012). For the core specialties, the BEES scores dropped while the students progressed through medical school but still remained in the “average” range as described by Mehrabian (1996). Yet among these five core specialties, there was a considerable amount of physician burnout (Shanafelt et al., 2012). Internal and family medicine physicians had burnout rates of 54 and 50%, respectively. Ob/Gyn was close behind with a 46% burnout rate; psychiatry and general pediatrics, which had the lowest burnout rate, fared better with burnout rates of 40 and 35%, respectively. Non-core specialties with BEES scores ranked as “slightly low” (−0.5 s.d. lower than the population norm) had burnout rates that ranged from the highest level of 65% (emergency medicine) to 45–40% (diagnostic radiology, general surgery, ophthalmology, urology, medical pediatrics). The two specialties ranked as “moderately low” (−1.0 s.d.), orthopedics and pathology, had burnout rates of 47 and 37%, respectively. When comparing these BEES data with JSPE scores, any core specialty having an “average” BEES score was associated with a JSPE score of over 120 (JSPE range: 20–140), whereas most remaining specialties had JSPE scores <120.

So how does a physician in a specialty with a high burnout rate still maintain an “average” amount of affective empathy? It's possible core physicians who are in the front line of primary care (family and internal medicine) are more efficient at using ToM and cognitive empathy skills to more effectively blunt their affective empathy so the burnout they are experiencing does not further decrease their average-ranked BEES scores into lower rankings which are −0.5 to −2.0 s.d. off the population norm. In other words, the core physicians with higher JSPE scores are presumably better able to maintain empathic role-playing communication with their patients, even though they have burnout rates at or above 50%. However, the conundrum is cognitive control over affective inputs takes an emotional toll on physicians and contributes to higher rates of burnout—especially for women (Lloyd and Gartrell, 1981; Hojat et al., 1999; Lindfors et al., 2009; Backović et al., 2012). Those physicians in non-core specialties, who theoretically do not need to display or use as many cognitive empathy skills with their patients, have cognitive empathy JSPE scores lower than physicians in core specialties. These non-core physicians may not feel the need to communicate effectively with their patients and therefore do not need to go through the emotional labor to role-play an empathic response to the patient. Clearly, additional research is needed to elucidate the interactions of affective and cognitive empathy with burnout and stress, especially regarding how a physician actually reacts to patients (i.e., state empathy) vs. their trait empathy.

## Should physicians have a hardened empathic heart?

Physicians frequently deal with the emotional burden of life, death, and patients in pain during their practice, yet still have to relate to patients in an empathic manner. There are several ways a physician can respond to this burden. A physician can be empathically neutral and perform what needs to be done to the patient without feeling grief, regret, or other difficult emotions. Alternatively, detached insight could be used to communicate with and treat the patient. This detachment, orchestrated by ToM and cognitive empathy, blunt the affective empathy pathways allowing the physician to respond to the patient with role-playing behavior. Accomplishing this may be more difficult than it sounds, since displaying role-playing empathy for the patient, while feeling affective empathy which is different from what you really want to express, leads to an empathic dissonance within the physician. It takes considerable effort for the physician to put forward an empathic front for the patient, especially when the physician has a negative emotional reaction to the patient that causes personal distress. Many physicians find maintaining an empathic relationship with patients is not an easy task and can be likened to an emotional labor. Just as one example, there are complex biopsychosocial interactions needed to interpret the degree of an individual's pain and to respond with an appropriate level of empathic support (Hadjistavropoulos et al., 2011).

So is it necessary for a physician to have a hardened heart? Being too empathetic can leave the medical personnel vulnerable to the negative consequences of a patient's medical condition (Badger et al., 2008). An over empathic physician risks over-identifying with their patients, whereby emotional responses from the patient can threaten medical objectivity. Therefore, a certain amount of emotional detachment from the patient is necessary or else the physician lets the affective empathy bring about feelings within themselves that detracts from their ability to effectively manage the medical situation. Yet, on the other end of the empathic spectrum, a total detachment from the patient by a physician who appears not to care or is callous, does not establish the empathic connection the patient desires and expects.

For those physicians entering core, patient-oriented specialties, maintaining an average level of affective empathy, while having higher cognitive empathy skills would be beneficial in maintaining a positive physician–patient rapport. However, this level of empathy would not necessarily benefit physicians entering non-core specialties, since they deal with patients with more intrusive techniques—even if ordered by a core physician. Allowing too much affective empathy to overwhelm non-core physicians as they perform surgeries, endoscopic exams, or diagnose patient pathologies, would potentially lead to ineffective treatment of the patient as the physician pays more attention to their own affective inputs vs. concentrating on the patient. Therefore, for the non-core specialty physician, having a lesser amount of affective empathy should result in less effort to maintain a reasonable detachment from the patient and enable more efficient patient care.

Ultimately, the answer to the question is—“Yes”—physicians need to harden their heart, but like most things in life the answer is not “black or white.” Empathic shades of gray are needed depending on the physician's specialty and their innate levels of affective and cognitive empathy. Assuredly, the most emotionally difficult task for the physician is to moderate the degree to which they harden their hearts. Physicians walk a fine empathic line to ensure they can relate to the patient without becoming too hardened themselves.

### Conflict of interest statement

The author declares that the research was conducted in the absence of any commercial or financial relationships that could be construed as a potential conflict of interest.
